# Checkpoint-Dependent and -Independent Roles of Swi3 in Replication Fork Recovery and Sister Chromatid Cohesion in Fission Yeast

**DOI:** 10.1371/journal.pone.0013379

**Published:** 2010-10-12

**Authors:** Jordan B. Rapp, Chiaki Noguchi, Mukund M. Das, Lisa K. Wong, Alison B. Ansbach, Allyson M. Holmes, Benoit Arcangioli, Eishi Noguchi

**Affiliations:** 1 Department of Biochemistry and Molecular Biology, Drexel University College of Medicine, Philadelphia, Pennsylvania, United States of America; 2 Unité de Dynamique du Génome, URA 1644 du CNRS, Departement de la Structure et Dynamique des Génomes, Institut Pasteur, Paris, France; St George's University of London, United Kingdom

## Abstract

Multiple genome maintenance processes are coordinated at the replication fork to preserve genomic integrity. How eukaryotic cells accomplish such a coordination is unknown. Swi1 and Swi3 form the replication fork protection complex and are involved in various processes including stabilization of replication forks, activation of the Cds1 checkpoint kinase and establishment of sister chromatid cohesion in fission yeast. However, the mechanisms by which the Swi1–Swi3 complex achieves and coordinates these tasks are not well understood. Here, we describe the identification of separation-of-function mutants of Swi3, aimed at dissecting the molecular pathways that require Swi1–Swi3. Unlike *swi3* deletion mutants, the separation-of-function mutants were not sensitive to agents that stall replication forks. However, they were highly sensitive to camptothecin that induces replication fork breakage. In addition, these mutants were defective in replication fork regeneration and sister chromatid cohesion. Interestingly, unlike *swi3*-deleted cell, the separation-of-functions mutants were proficient in the activation of the replication checkpoint, but their fork regeneration defects were more severe than those of checkpoint mutants including *cds1*Δ, *chk1*Δ and *rad3*Δ. These results suggest that, while Swi3 mediates full activation of the replication checkpoint in response to stalled replication forks, Swi3 activates a checkpoint-independent pathway to facilitate recovery of collapsed replication forks and the establishment of sister chromatid cohesion. Thus, our separation-of-function alleles provide new insight into understanding the multiple roles of Swi1-Swi3 in fork protection during DNA replication, and into understanding how replication forks are maintained in response to different genotoxic agents.

## Introduction

A variety of agents, including environmental toxins or drugs, can cause DNA damage and lead to arrest of DNA replication forks. Arrested forks are among the most serious threats to genomic integrity because they can collapse, break, or rearrange [1], [2], [3]. To circumvent these problems, cells are equipped with a DNA replication stress response pathway, termed the DNA replication checkpoint or the S-phase checkpoint. This checkpoint is activated by impeded replication forks and arrests the cell cycle while reducing the rate of DNA synthesis in order to coordinate with DNA repair and preserve genomic integrity [4], [5], [6].

In the fission yeast *Schizosaccharomyces pombe*, atop the replication checkpoint system stands a protein kinase, Rad3, which is homologous to human ATM and ATR [7], [8], [9]. Rad3 controls downstream effector kinases Cds1 (functional homolog of human Chk1) and Chk1 (functional homolog of human Chk2), both of which are also conserved throughout evolution [7], [8], [9]. Chk1 promotes the DNA damage checkpoint pathway while Cds1 acts as the master kinase for activation of the replication checkpoint to phosphorylate Cdc25, thereby inhibiting the Cdc2 (Cdk1) kinase and facilitating DNA repair and recombination pathways [7], [8], [9], [10], [11], [12], [13]. Another important function of the replication checkpoint is to stabilize replication forks by maintaining proper assembly of replisome components and preserving DNA structures when problems are encountered during DNA replication [14], [15], [16], [17], [18]. In fission yeast, we have demonstrated that Cds1 prevents fork collapse in response to hydroxyurea (HU) [19], a compound that arrests replication forks, indicating that Cds1 is required for stabilization of stalled replication forks in a replication competent state. However, the precise molecular mechanisms by which stalled forks activate the replication checkpoint are not completely understood.

In our previous studies concerning the mechanisms of the replication checkpoint, we found that Swi1 is required for proper activation of Cds1 in response to HU and for stabilization of replication forks in fission yeast [19]. Further investigation has revealed that Swi1 interacts with Swi3 and travels with the replication fork as a replisome component [20]. In the absence of Swi1 or Swi3, cells accumulate Rad22 DNA repair foci in S-phase [19], [20]. These foci correlate with the Rad22-dependent appearance of Holliday junction (HJ)-like structures [20]. Rad22 is a Rad52 homolog and is known to bind single-stranded DNA (ssDNA) regions at the site of DNA damage [21], [22]. Thus, our results suggest a high rate of fork abnormalities in *swi1Δ* and *swi3Δ* mutant cells, generating ssDNA regions near the replication fork, which induces accumulation of HJ-like structures [19], [20]. Based on our results, we have referred to the Swi1–Swi3 complex as “the Replication Fork Protection Complex” (FPC) [20]. The Swi1–Swi3 complex is evolutionarily conserved and is homologous to the Tof1-Csm3 complex in *Saccharomyces cerevisiae* and the Timeless-Tipin complex in humans [20], [23], [24], [25], [26]. Tof1-Csm3 has been shown to be part of the replisome or the replisome progression complex (RPC) and is involved in Rad53 activation [27], [28], [29], [30]. In humans, Timeless-Tipin interacts with Chk1 and ATR to control activation of checkpoint kinase Chk1 [31], [32], [33], [34]. We have also demonstrated that Timeless-Tipin moves with replication forks, functions to stabilize replication forks, and facilitates sister chromatid cohesion in human cells [35]. However, it remains unclear how Swi1–Swi3 related complexes interact with and stabilize replication forks and coordinate with multiple genome maintenance processes. Therefore, it is important to understand the functions of Swi1–Swi3, by dissecting molecular pathways that require this protein complex.

In the present studies, we have carried out a mutational analysis of *S. pombe* Swi3 to further understand the functions of the Swi1–Swi3 replication fork protection complex. We identified separation-of-function mutations of Swi3, which leads us to propose that Swi3 utilizes different molecular mechanisms to regulate the replication checkpoint and sister chromatid cohesion. Swi3 appears to use the replication checkpoint pathway to stabilize stalled replication forks. However, when broken forks are present, Swi3 functions to restore forks using a checkpoint-independent pathway, which is also important for proper establishment of sister chromatid cohesion.

## Results

### Isolation of *swi3* mutants

**Figure 1:**
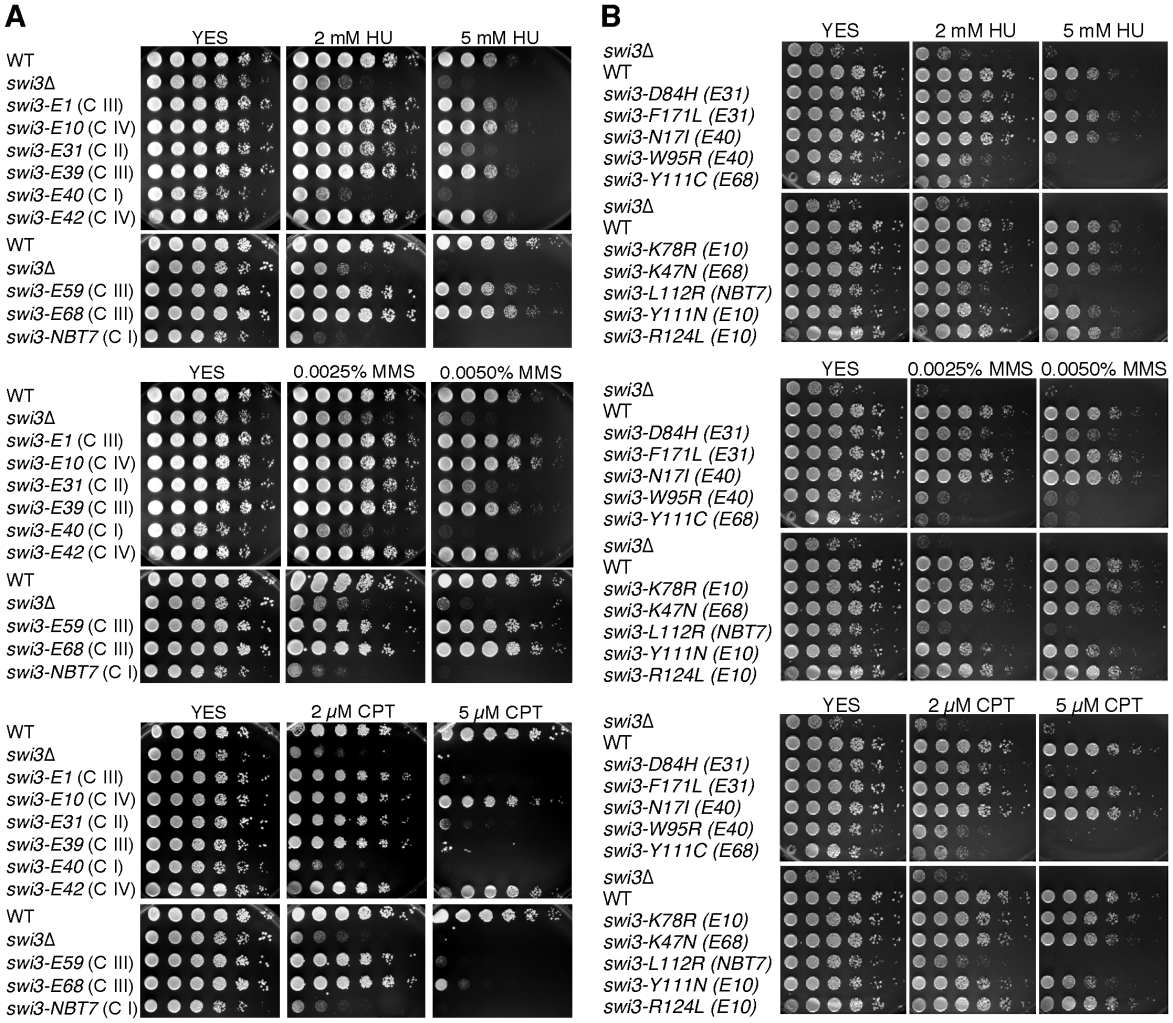
Sensitivity of *swi3* mutants to S-phase stressing agents. (**A, B**) Five-fold serial dilutions of cells of the indicated genotypes were incubated on YES agar medium supplemented with the indicated amounts of HU (top panels), MMS (middle panels) and CPT (bottom panels) for 3 to 5 days at 32°C. In A, classes (C I to C IV) of *swi3* mutants are indicated in parentheses. In B, original *swi3* alleles from which the single point mutations were derived are also indicated in parentheses. Representative images of repeat experiments are shown.

To understand the roles of the Swi1–Swi3 complex in the S-phase stress response, we isolated a number of *swi3* mutants using error prone PCR (*swi3* E-series). The wild-type *swi3* gene was replaced with mutagenized *swi3-5FLAG* genes at the *swi3* genomic locus, and mutants were tested for their viability in YES medium containing a high dose of hydroxyurea (HU, 10 mM) or camptothecin (CPT, 10 µM). HU depletes the dNTP pool and causes an arrest of replication fork progression, while CPT traps topoisomerase I on DNA and induces replication fork breakage. Among 20 HU and/or CPT-sensitive mutants, 12 mutants failed to express Swi3 as a 5FLAG fusion protein, suggesting that these mutants contain non-sense or frame-shift mutations that cause early termination of Swi3 translation (data not shown). Therefore, we decided to further characterize the remaining 8 mutants and *swi3-NBT7*, which was individually isolated as a mating-type switching defective mutant (see Materials and Methods). These mutants were more carefully examined for sensitivities to HU and CPT. For sensitivity assays, we also included methyl methanesulfonate (MMS), which causes replication fork arrest by alkylating template DNA. The 9 mutants were categorized into four groups according to their drug sensitivity. Class I mutants (*swi3-E40* and *NBT7*) showed strong sensitivity to 2 mM HU, 0.0025% MMS and 2 µM CPT (Figure 1A), which was comparable to that of *swi3*Δ cells. Class II mutant (*swi3-E31*) was sensitive to 5 mM HU, 0.005% MMS and 5 µM CPT (Figure 1A). Class III mutants (*swi3-E1*, *E39, E59* and *E68*) were not significantly sensitive to HU and MMS, but did show significant sensitivity to 5 µM CPT (Figure 1A). Class IV mutants (*swi3-E10*, and *E42*) were only sensitive to HU, MMS or CPT at very high doses (10 mM HU, 0.01% MMS and 10 µM CPT, data not shown) where wild-type cells start to decrease their viability. Drug sensitivities of *swi3* mutants are summarized in Table 1.

**Table 1:** Summary of *swi3* mutants characterized in this study.

			Growth rate						
Strain	Class	Mutation(s)	YES	YES HU	YES MMS	YES CPT	Swi1 interaction	Cds1 activity	Rad22 foci
wild-type		none	+++	+++	+++	+++	+++	++++	−
*swi3*Δ		deletion	+++	−	−	−	N/A	+	+++
*swi3-E1*	III	R125H,A170V	+++	+++	+++	+	+++	++++	N/D
*swi3-E10*	IV	K78R,Y111N,R124L	+++	+++	+++	+++	+++	++++	N/D
*swi3-E31*	II	D84H, F171L	+++	+	+	+	−	+++	+
*swi3-E39*	III	W128R	+++	+++	+++	+	+++	++++	+/−
*swi3-E40*	I	N17I, W95R	+++	−	−	−	−	++	+++
*swi3-E42*	IV	M91I	+++	+++	+++	+++	+++	++++	N/D
*swi3-E59*	III	I94K,K68E,D177N	+++	+++	+++	+	+++	++++	N/D
*swi3-E68*	III	K47N,Y111C	+++	+++	+++	+	+++	++++	N/D
*swi3-NBT7*	I	L112R	+++	−	−	−	−	+	+++
*swi3-D84H*		D84H	+++	+	+	+	−	N/D	+++
*swi3-F171L*		F171L	+++	+++	+++	+++	+++	N/D	+
*swi3-N17I*		N17I	+++	+++	+++	+++	+++	N/D	+
*swi3-W95R*		W95R	+++	−	−	−	−	N/D	+++
*swi3-Y111C*		Y111C	+++	−	−	−	−	N/D	+++
*swi3-K78R*		K78R	+++	+++	+++	+++	+++	N/D	+/−
*swi3-K47N*		K47N	+++	+++	+++	+++	+++	N/D	+
*swi3-L112R*		L112R	+++	−	−	−	−	N/D	+++
*swi3-Y111N*		Y111N	+++	+++	+++	+++	+++	N/D	+/−
*swi3-R124L*		R124L	+++	+++	+++	+++	+++	N/D	+

### Effects of *swi3* mutations on the formation of the Swi1–Swi3 complex

**Figure 2:**
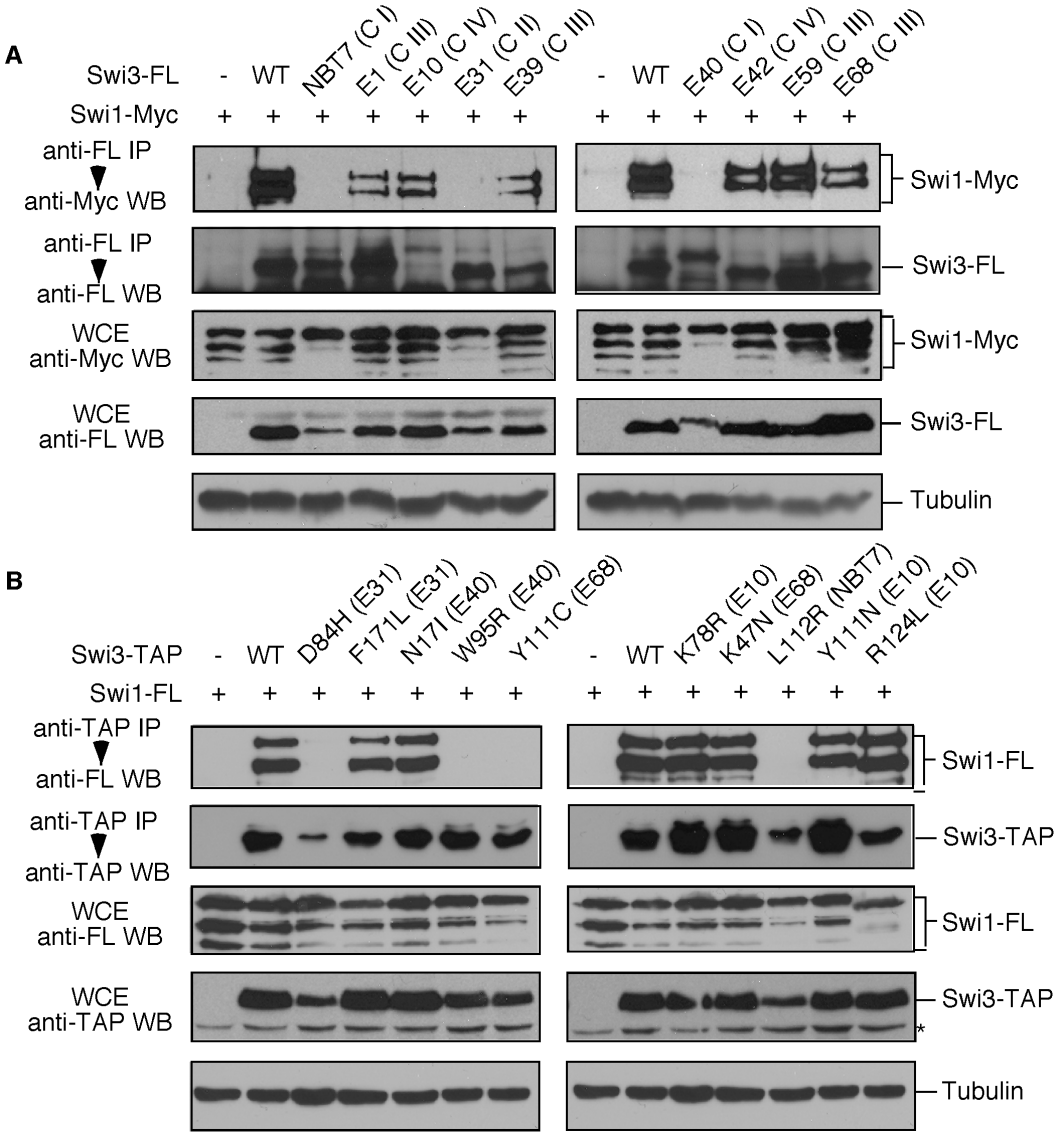
Effects of *swi3* mutations on the formation of the Swi1-Swi3 complex. (**A**) Protein extracts were prepared from cells expressing the indicated fusion proteins. Swi3-FLAG (Swi3-FL) was precipitated, and associated proteins were probed with the anti-Myc 9E10 and anti-FLAG M2 antibodies. Classes (C I to C IV) of Swi3 mutants are indicated in parentheses. The appearance of two to three bands in Swi1-Myc Western blots is due to degradation of the fusion protein [20], [24]. The Swi3-E40 mutant protein showed slower mobility, which is possibly due to mutational effects. Although only small amount of the Swi3-E10 protein was recovered by immunoprecipitation, Swi1-13Myc was efficiently co-precipitated with Swi3-E10. Western blotting of tubulin was performed as a loading control. (**B**) Protein extracts from the indicated strains were subjected to Swi3-TAP precipitation experiments, and associated proteins were probed with the anti-FLAG M2 and PAP antibodies. Original *swi3* alleles from which the single point mutations were derived are also indicated in parentheses. Although reduced amount of Swi3 were recovered by immunoprecipitation in *swi3-D84H*, *L112R* and *R124R*, they were all readily detected. Asterisk indicates non-specific bands. Representative results of repeat experiments are shown. IP, immunoprecipitation; WB, Western blotting; WCE, whole cell extract.

Swi1 is known to co-purify with Swi3 from *S. pombe* cell extracts [20], [24]. Therefore, to address the effect of Swi3 mutations on Swi1–Swi3 complex formation, we performed immunoprecipitation assays to examine the ability of the Swi3 mutant proteins to interact with Swi1. Cells expressing Swi3-5FLAG mutant proteins were engineered to produce Swi1-13Myc from its genomic locus. As shown in Figure 2A, all mutant cells expressed Swi1-13Myc and Swi3-5FLAG proteins from their endogenous promoters. Swi1-13Myc consistently showed a series of degraded bands possibly due to proteolysis at specific sites in Swi1 (Figure 2). Interestingly, *swi3-E31*, *E40* and *NBT7* mutant cells reproducibly expressed reduced amounts of the Swi3 protein compared to *swi3* ^+^ cells, although they are readily detectable (Figure 2A). Accordingly, Swi3-5FLAG was immunoprecipitated, and Swi1 associated with Swi3 was examined by immunoblotting using the anti-FLAG and Myc antibodies. As shown in Figure 2A, considerable amounts of Swi3 mutant proteins were recovered from all mutants except for *swi3-E10*. Although the amount of Swi3 recovered from *swi3-E10* cells was much less than other mutants, it was still detectable. Notably, there was no detectable interaction of Swi1-13Myc and Swi3-5FLAG in *swi3-E31*, *E40* and *NBT7* (Classes I and II) cells, whereas other mutants retained significant levels of Swi1–Swi3 complex formation (Figure 2A). Considering that *swi3-E31*, *E40* and *NBT7* are significantly sensitive to HU, MMS and CPT (Figure 1A and Table 1), these data suggest that Swi1–Swi3 complex formation is required for tolerance to replication fork arrest and damage. We also observed that *swi3-E1*, *E39*, *E59* and *E68* (Class III), which retained Swi1–Swi3 complex formation, were only sensitive to CPT (Figures 1A and 2A); suggesting that CPT sensitivity is not caused uniquely by a defect of formation of the Swi1–Swi3 complex, and that Swi1–Swi3 possesses at least two separate functions in the preservation of genomic integrity.

**Figure 3:**
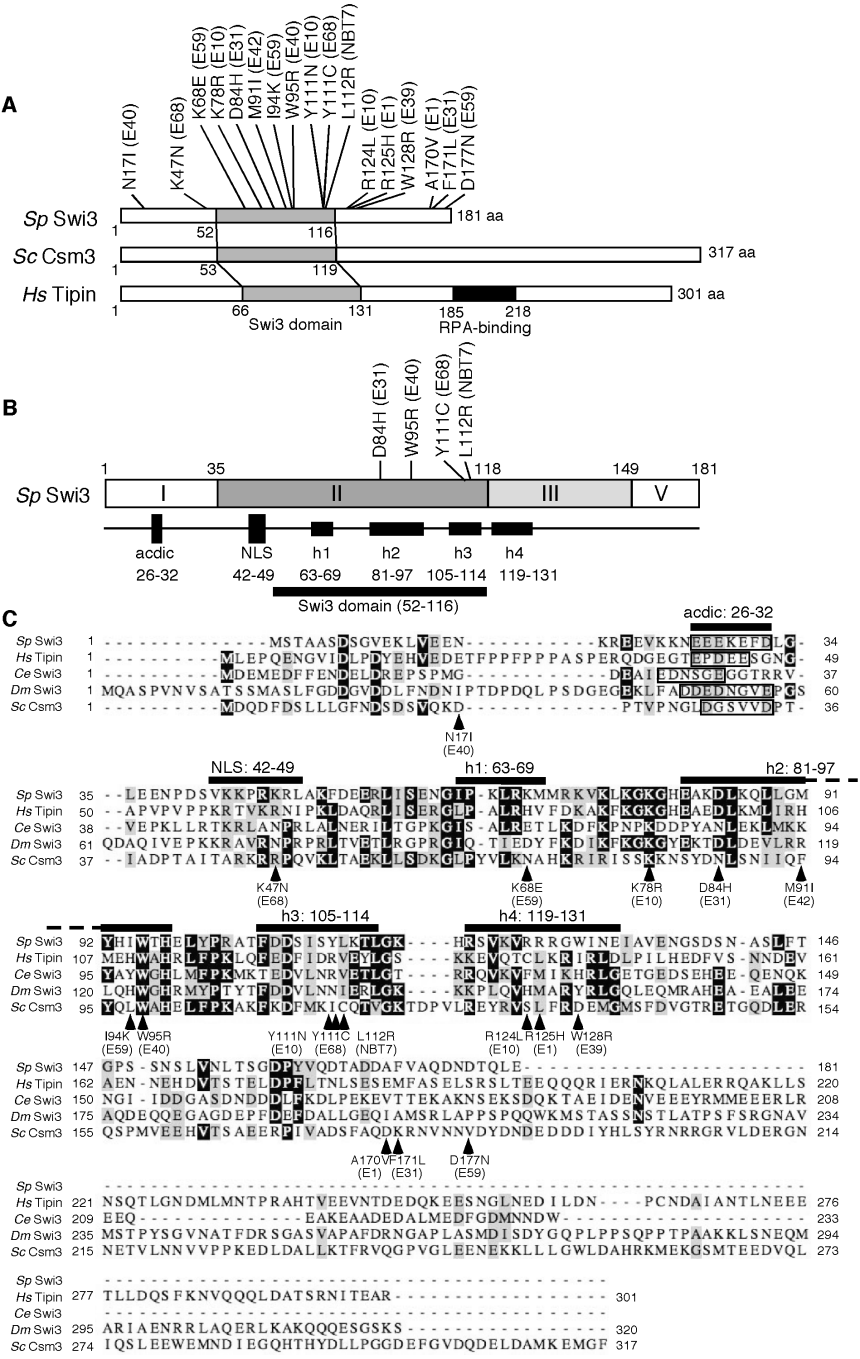
Structure of Swi3 related proteins. (**A**) Schematic drawing of Swi3 homologs from *S. pombe* (*Sp* Swi3), *S. cerevisiae* (*Sc* Csm3) and humans (*Hs* Tipin). Gray boxes indicate regions of amino acid sequences that are highly conserved throughout evolution. This region in each protein is called the Swi3 domain. The RPA-binding motif is found only in human Tipin. Mutation sites found in *swi3* alleles are indicated. aa, amino acid. (**B**) The Swi3 polypeptide was divided into 4 putative functional sub-domains. The dark gray box (Domain II) indicates the region with amino acid sequences that are conserved throughout evolution. This region contains a putative NLS (42–49 aa) and the Swi3 domain (52–116 aa), the latter of which includes three conserved α-helices: h1 (63–69 aa), h2 (81–97), and h3 (105–114 aa). The light gray box (Domain III) has amino acid sequences that are weakly conserved among species and contain a conserved α-helix (h4, 119–131 aa). Swi3 also has a stretch of acidic amino acids at 26–32 within Domain I. The positions of mutations that abolish Swi1–Swi3 complex formation are indicated. aa, amino acid. (**C**) Multiple sequence alignment of Swi3 homologs from *S. pombe* (Sp Swi3), humans (Hs Tipin), *C. elegans* (Ce Swi3), *Drosophila melanogaster* (Dm Swi3) and *S. cerevisiae* (Sc Csm3). Locations of the putative NLS, the conserved acidic region, the conserved α-helices, and mutations found in our *swi3* mutant collection are shown.

DNA sequencing analysis of *swi3* mutants isolated by error prone PCR (*swi3-E* series) revealed that many of them contained multiple mutations in *swi3* (Table 1). Therefore, we employed site-directed mutagenesis to introduce single-point mutations at sites found in *swi3-E10*, *swi3-E31*, *swi3-E40*, and *swi3-E68* (Table 1). These mutants and *swi3-NBT7* (L112R) were expressed from the *swi3* promoter as TAP fusion proteins in *swi3*Δ *swi1-3FLAG* cells. As shown in Figure 2B, *swi3-D84H* (from *swi3-E31*), *swi3-W95R* (from *swi3-E40*), and *swi3-L112R* (from *swi3-NBT7*) mutant cells expressed somewhat lower amounts of Swi3-TAP protein. Moreover, Swi3-D84H, Swi3-W95R, and Swi3-L112R proteins failed to interact with Swi1 (Figure 2B). These data are consistent with the results of the original mutants (*swi3-E* series) that showed strong sensitivity to genotoxic agents (Figure 1A and 2A, and Table 1). Interestingly, when the two mutations (Y111C and K47N) present in Swi3-E68 were characterized individually, we found that the expression level of Swi3-Y111C was lower than wild-type and that Swi3-Y111C failed to interact with Swi1 (Figure 2B). In contrast, Swi3-K47N expression and its ability to interact with Swi1 were indistinguishable from the wild-type Swi3 protein (Figure 2B). We obtained similar results when the single-point Swi3 mutants were expressed as FLAG-fusion proteins (data not shown). Taken together with the fact that the original mutant (Swi3-E68) retained ability to interact with Swi1 (Figure 2A), these results suggest that the conformational change induced by Y111C abolishes the interaction with Swi1, which is compensated by the K47N mutation. More importantly, all of the single-point mutations that eliminate Swi1–Swi3 complex formation are located within the central “Swi3 domain” region (52–116 amino acids), which shows significant homology throughout evolution (Figures 3A) [20]. Consistently, Swi3-D84H, W95R, Y111C and L112R mutants were all highly sensitive to HU, MMS and CPT (Figure 1B), suggesting that complex formation is important for cellular tolerance to S-phase stressing agents.

### Structural prediction of Swi3

To understand the molecular basis of the Swi1-Swi3 replication fork protection complex, we performed structural analyses of the Swi3 protein at the amino acid sequence level. We used ClustalW multiple Sequence Alignment of Swi3-related proteins, including human Tipin, *Drosophila* Swi3 (dmSwi3), *C. elegans* Swi3 (ceSwi3), *S. pombe* Swi3 and *S. cerevisiae* Csm3. This analysis predicted that dmSwi3 and Csm3 have stretches of amino acid sequences that may divide Swi3-related proteins into at least 4 functional domains (Figure 3B and 3C). The N-terminal domain (Domain I: 1–34 amino acids) had weak similarity among the species and contained acidic amino acid-rich sequences. The central domain (Domain II: 35–117 amino acids) possessed significant homology throughout evolution. We have also found a putative nuclear localization signal (NLS: 42–49 amino acids) using the PredictNLS program provided by Columbia University. Although the NLS was only found in *S. pombe* Swi3, the corresponding regions from other species were rich in basic amino acids. Interestingly, using the Jpred3 secondary structure prediction program provided by University of Dundee, we found that Domain II contained three alpha helices, which were also conserved among the species. Although, the third domain (Domain III: 118–149 amino acids) was only weakly conserved, Jpred3 found that N-terminal part of this domain contained a conserved alpha helix structure. The fourth domain (Domain IV: 150–181 amino acids) appeared not to be conserved and varied in their length between species. Interestingly, the RPA-binding motif found in mammalian Tipin [25], [33] was not conserved in *S. pombe*, *S. cerevisiae*, *C. elegans* and *Drosophila* (Figure 3A and 3C). It is important to note that all the mutations that disrupted Swi1–Swi3 complex formation (D84H, W95R, Y111C and L112R) were found in one of the alpha helices within the central conserved Swi3 domain, suggesting that alpha helix structures in Domain II play a role in interacting with Swi1 (Figure 3B and 3C).

### Cellular phenotypes of *swi3* mutants

**Figure 4:**
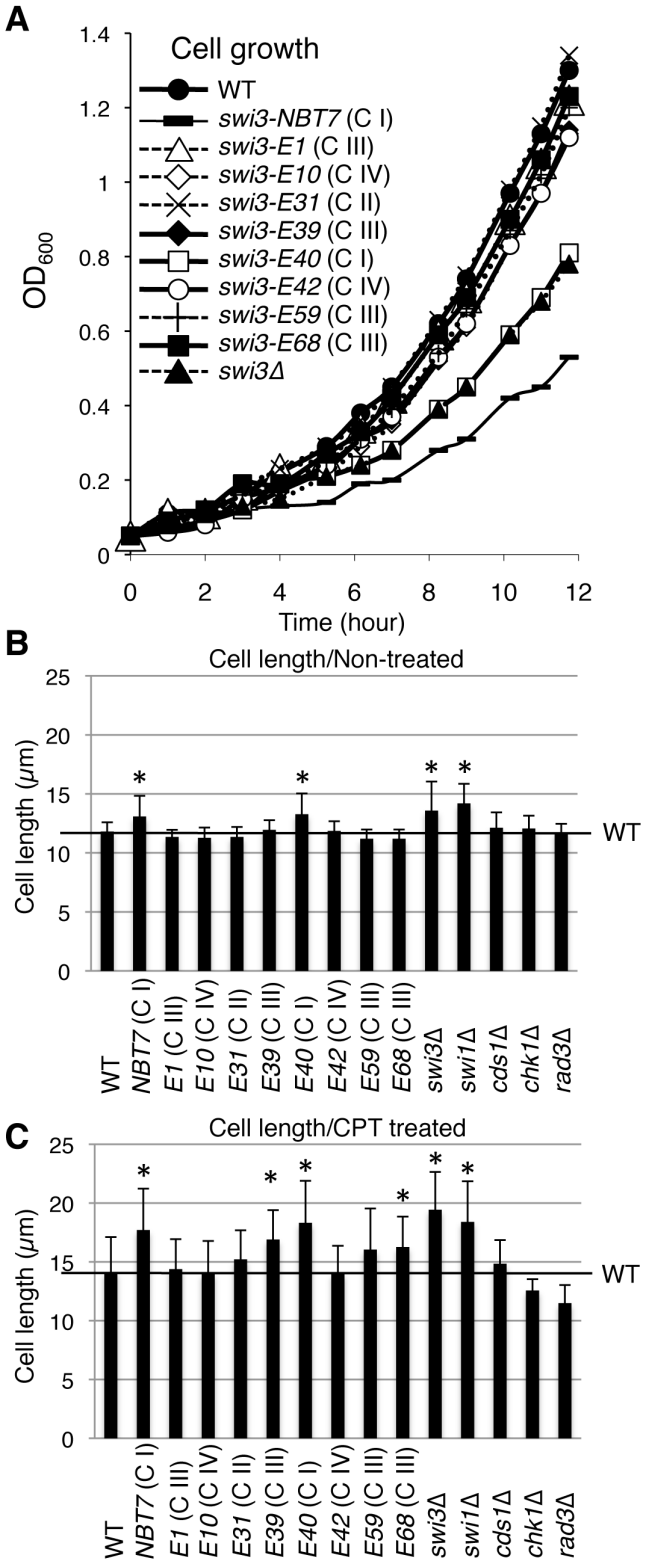
Effects of *swi3* mutations on cell growth and length. (**A**) Cells of the indicated genotypes were grown in YES media at 30°C and measured for OD_600 nm_ values at the indicated times. (**B, C**) Cells of the indicated genotypes were grown in YES supplemented with 0 (B) or 30 µM (C) CPT for 7 h at 25°C, and cell length at septation was measured. At least 25 septated cells were measured for each strain. Error bars correspond to standard deviations. * *P*-values (<0.01) determined by paired Student's *t*-test indicate that these mutants show statistically significant elongation phenotype compared to wild-type cells.

We have previously shown that *swi1*Δ and *swi3*Δ cells are moderately elongated with mild growth defect and that this mitotic delay requires Chk1 but not Cds1 [19], [20]. Therefore, we determined growth rates and cell lengths of *swi3* mutants. The growth rates of *swi3-E1*, *E10*, *E31*, *E39*, *E42*, *E59* and *E68* (Classes II, III and IV) cells were comparable to that of wild-type cells, whereas *swi3-E40* (Class I) showed mild growth defects similar to *swi3*Δ (Figure 4A). Interestingly, *swi3-NBT7* (Class I) had slower growth rate than *swi3*Δ (Figure 4A). Consistent with these results, *swi3-E40* and *NBT7* cells (Class I) showed moderate but statistically significant cell elongation phenotype in the absence of genotoxic agents, which was similar to that of *swi1*Δ and *swi3*Δ (Figure 4B). We then treated *swi3* mutants with CPT and measured their dividing cell length (Figure 4C). Wild-type cells showed mild elongation, probably due to a cell cycle delay provoked by replication fork breakage (Figure 4B and 4C. non-treated: 11.80 µm; CPT-treated: 14.12 µm; *p*-value = 0.0013). Consistent with the fact that CPT activates the Chk1-dependent checkpoint pathway [36], *chk1*Δ cells failed to show a significant elongation phenotype (non-treated: 12.07 µm; CPT-treated: 12.57 µm). Rad3, which is known to activate Chk1, also appear to be important for this cell cycle delay (non-treated: 11.76 µm; CPT-treated: 11.50 µm). In contrast, *cds1*Δ cells showed mild elongation phenotype similar to wild-type (non-treated: 12.13 µm; CPT-treated: 14.84 µm; *p*-value = 0.0006), indicating that Cds1, a master kinase required for the replication checkpoint, does not have a major role in CPT-dependent cell cycle delay. When treated with CPT, Class I mutants (*E40* and *NBT7*) were significantly more elongated than wild-type cells. This elongation was similar to that of *swi3*Δ and *swi1*Δ cells (Figure 4C), suggesting that Class I mutant cells experience severe difficulty in recovering broken replication forks. Class IV mutants (*E10* and *E42*) were similar to wild-type. However, in response to CPT, *swi3-E39* (Class III) and *E68* (Class III) also displayed statistically stronger elongation phenotype, and *swi3-E31* (Class II) and *E59* (Class III) reproducibly showed somewhat more elongated phenotype when compared to wild-type. These results suggest that Class II and III mutants might have difficulty in recovering broken replication forks after CPT exposure, and they are consistent with the camptothecin sensitivity of the *swi3* mutants (Figure 1C).

### Effects of *swi3* mutations on the recovery of broken replication forks

**Figure 5:**
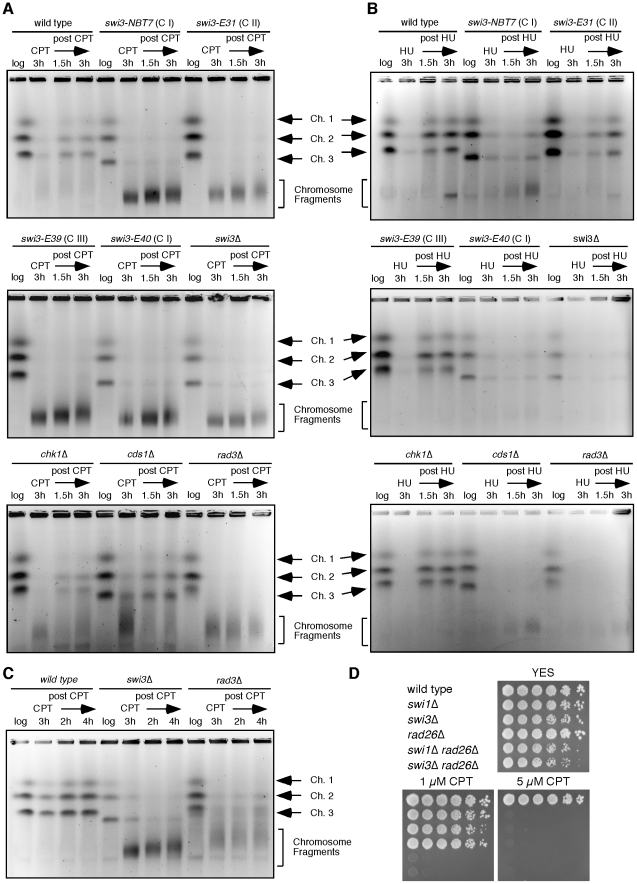
Effects of *swi3* mutations on the recovery of replication forks. (**A, B, C**) Chromosome samples from cells of the indicated genotypes were examined by PFGE. Cells were grown until mid-log phase and then incubated in the presence of 30 µM CPT (A), 20 mM HU (B) or 15 µM CPT (C) for 3 h at 30°C. Cells were then washed and released into fresh medium. Chromosomal DNA samples were prepared at the indicated times. *swi3* (except for *swi3-E39*) and *cds1* mutants appeared to harbor a shorter chromosome III, which is probably due to recombination at rDNA repeats [23], [37], [66]. Representative results from repeat experiments are shown. (D) Five-fold serial dilutions of cells of the indicated genotypes were incubated on YES agar medium supplemented with the indicated amounts of CPT for 3 days at 32°C.

We have previously shown that Swi1 and Swi3 are required for stabilization of replication forks [19], [20], [37]. To investigate the effect of Swi3 mutations on replication fork stability, we examined the recovery of DNA replication after fork breakage induced by CPT treatment. We chose representative *swi3* mutant(s) from each *swi3* mutation class, including *swi3-NBT7* and *swi3-E40* (Class I), *swi3-E31* (Class II), and *swi3-E39* (Class III). Class IV mutants were not included because they were not significantly sensitive to genotoxic agents (Figure 1). Chromosome samples of wild-type and *swi3* mutant cells were prepared before and at 3 h after CPT treatment, and at different time points during recovery after the removal of CPT. These chromosomes were then resolved by PFGE, which allows only a fully replicated chromosomes to appear in the gel (Figure 5A, the top and middle panels). Intact chromosomes from exponentially growing cells (log) in wild-type and all mutant strains migrated into the gel. CPT treatment causes replication fork breakage, leading to the reduction in the amount of intact chromosomes that migrated into the gel in wild-type and all *swi3* mutant cells. When cells were returned into fresh medium without CPT, intact chromosomes from wild-type cells re-appeared in the gel at 1.5 h after CPT removal due to the completion of DNA synthesis. However, intact chromosomes from all *swi3* mutant cells failed to migrate into the gel at 1.5 h and 3 h during recovery, indicating that Swi3 is required for the recovery of DNA replication after fork breakage. In addition, all *swi3* mutants contained excessive amounts of fragmented chromosomes during and after CPT exposure (Figure 5A, the top and middle panels), suggesting that Swi3 might be involved in efficient repair of broken replication forks.

### Swi3 plays a role in recovery of broken replication forks in a manner independent of checkpoints

It is known that Swi3 is important for efficient activation of the Cds1-dependent replication checkpoint [20]. Therefore, we compared the replication recovery defect of *swi3* mutants with that of checkpoint mutants (Figure 5A, the bottom panel). *cds1*Δ cells failed to show significant defects in replication recovery after CPT exposure, indicating that Cds1 does not have a major role in the recovery of broken replication forks. It is known that Chk1 has a major function in the DNA damage checkpoint but also plays a redundant role with Cds1 in DNA replication checkpoint [12]. When *chk1*Δ cells were tested, a mild defect in replication recovery was observed in response to CPT. This is consistent with the fact that CPT activates the Chk1-dependent DNA damage checkpoint [36]. However, *chk1*Δ cells were able to recover replication more efficiently than any of the *swi3* mutants tested. In addition, there was much less accumulation of CPT-dependent fragmented chromosomes in both *cds1*Δ and *chk1*Δ cells compared to the *swi3* mutants. We also examined chromosomal DNA isolated from *rad3*Δ cells (Figure 5A, the bottom panel). *rad3*Δ cells failed to recover replication and accumulated fragmented chromosomes as expected from the role of Rad3 in activation of both Cds1 and Chk1. These results suggest that the replication checkpoint function of Swi3 does not have a major role in the recovery of broken replication forks induced by CPT. To further address this possibility, we directly compared *swi3*Δ and *rad3*Δ cells in the recovery of broken forks, using a lower dose of CPT and longer recovery time points (Figure 5C). In this condition, *rad3*Δ cells were able to recover broken replication forks more efficiently than *swi3*Δ cells (Figure 5C). In addition, *swi3*Δ cells accumulate significantly more fragmented DNA during recovery when compared to *rad3*Δ cells. Furthermore, *swi3*Δ *rad26*Δ cells were much more sensitive to CPT than either single mutant (Figure 5D). We also obtained similar results with *swi1*Δ *rad26*Δ cells in a CPT sensitivity assay (Figure 5D). Rad26 is essential for activation of Rad3, which is required for both Cds1 and Chk1 activities [38], [39]. Therefore, our results suggest that Swi3 has a specific role in replication recovery after fork breakage, which is independent of Cds1 or Chk1 activation.

### Effects of *swi3* mutations on the replication checkpoint

The Cds1-dependent replication checkpoint is required for the resumption of stalled replication forks in response to HU [9], [13], [19]. Since Swi3 is important for the full activation of Cds1 and for the stabilization of stalled replication forks in response to HU that activates Cds1 [20], we also monitored replication recovery after fork arrest due to HU exposure. *swi3-NBT7* (Class I), *swi3-E31* (Class II), *swi3-E39* (Class III), and *swi3-E40* (Class I) cells were treated with HU for 3 h and released into fresh medium to allow resumption of replication. As expected, *swi3*Δ, *cds1*Δ and *rad3*Δ cells, which all have defects in Cds1 activation, were not able to properly resume stalled forks after HU exposure (Figure 5B). *swi3-NBT7* and *E40* cells also showed resumption defects similar to *swi3*Δ (Figure 5B), suggesting the failure in Cds1 activation in these mutants. *swi3-E31* had mild defect in recovery from HU, which is consistent with its mild sensitivity to HU (Figure 1). Interestingly, *swi3-E39* cells were able to resume replication (Figure 5B) at the wild type level, suggesting that the Cds1-dependent replication checkpoint is still functional in this mutant.

**Figure 6:**
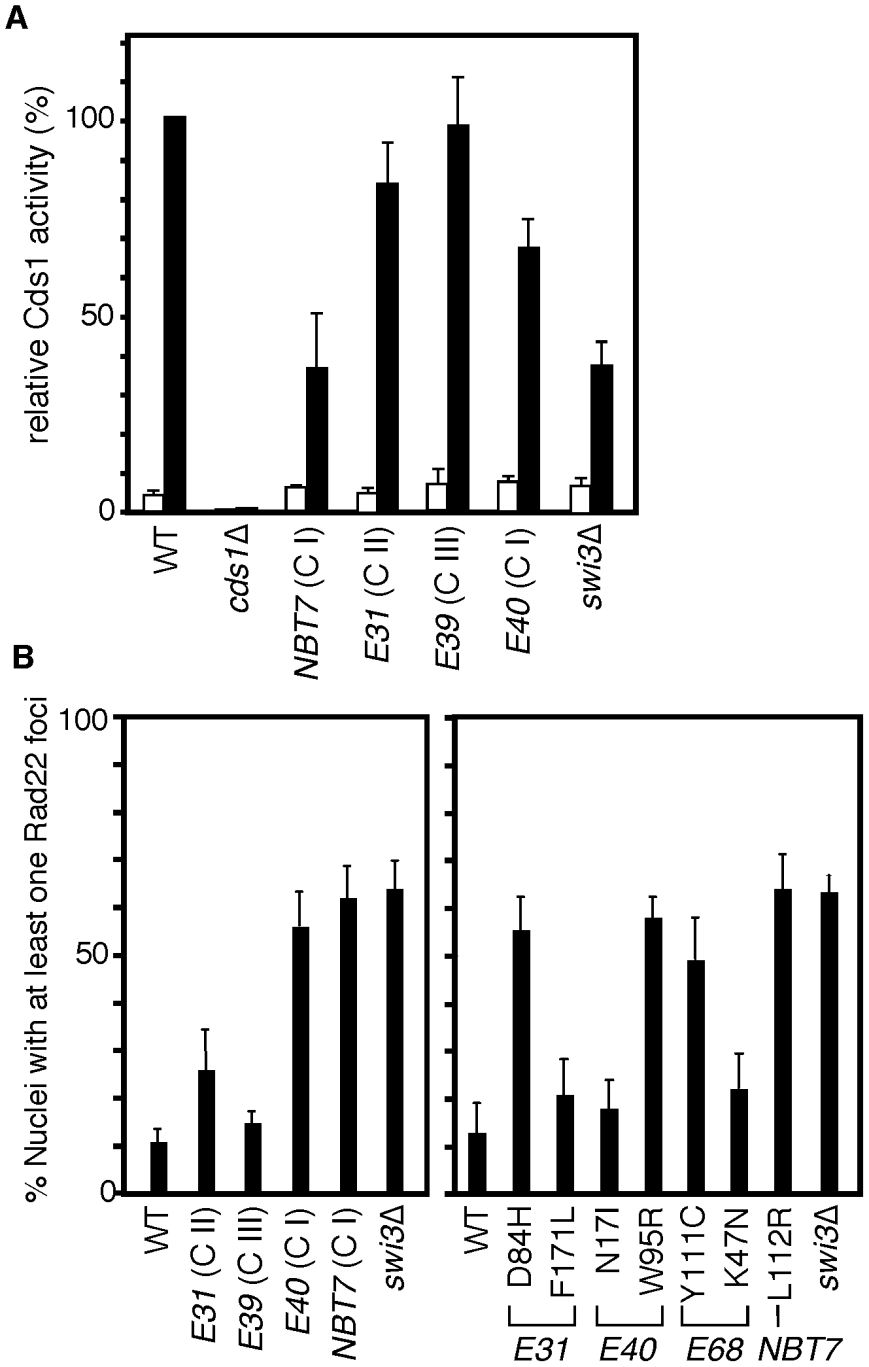
Effects of *swi3* mutations on Cds1 kinase activity and DNA repair foci formation. (**A**) Cells of the indicated genotypes were incubated in YES medium supplemented with 12 mM HU for 0 (open bars) and 2 h (closed bars) at 30°C. Kinase activity of immunoprecipitated Cds1 was measured using myelin basic protein (MBP) as a substrate. MBP was separated on 15% polyacrylamide gels and detected by Coomassie Brilliant Blue staining. The gel was dried, and radioactivity levels (cpm) of MBP were determined in a liquid scintillation counter. Relative radioactivity levels of Cds1 were calculated by setting the radioactivity of MBP from the HU-treated wild type sample to 100%. Error bars correspond to standard deviations obtained from three independent experiments. (**B**) Cells of indicated *swi3* mutants were engineered to express Rad22-YFP and grown in YES medium at 25°C until mid-log phase. The percentages of nuclei with at least one focus are shown. At least 200 cells were counted for each strain. Error bars correspond to standard deviations obtained from at least three independent experiments. This analysis shows that a large increase in Rad22-YFP foci accumulation was observed in *swi3* mutants that have a defect in Swi1–Swi3 complex formation.

Taken together, our present data indicate that Swi3 has a replication function that is independent of Cds1 activation. Therefore, we have examined the effects of *swi3* mutations on Cds1 activity. As shown in Figure 6A and Table 1, although there was a variation, Class I *swi3* mutants (*swi3-NBT7* and *E40*) had the most significant defects in Cds1 activation, which is consistent with the results of PFGE after HU treatment (Figure 5B). Class II mutants (*swi3-E31*) also displayed a slight decrease in Cds1 activation. However, Class III (*swi3-E1, E39, E59* and *E68*) and Class IV (*swi3-E10* and *E42)* appeared to have wild-type levels of Cds1 activity (Figure 6A and Table 1). These results indicate that Class I and II mutants but not Class III and IV mutants have a defect in the Cds1-dependent replication checkpoint. Taken together with the fact that *swi3-E39* (Class III) mutants failed to recover replication after fork collapse provoked by CPT (Figure 5A, middle panel), our results also indicate that Swi3's role in Cds1 activation is independent of the function of Swi3 in the recovery of broken replication forks.

### Replication abnormalities in *swi3* mutants

To further evaluate replication abnormalities in *swi3* mutants, we also monitored the formation of Rad22-YFP DNA repair foci in the absence of genotoxic agents. Rad22 is a homolog of budding yeast Rad52 and has been shown to bind ssDNA at the site of DNA damage [21], [22]. Depletion of *swi3* was shown to be associated with replication fork abnormalities, resulting in the strong accumulation of spontaneous Rad22-YFP DNA repair foci during unperturbed S-phase [20] (Figure 6B). Therefore, we monitored the formation of spontaneous Rad22-YFP foci in the *swi3* mutants. As shown in Figure 6B, we observed dramatically elevated levels of Rad22-YFP foci formation in *swi3-NBT7* (Class I) and *swi3-E40* (Class I) and significantly increased levels in *swi3-E31* (Class II) (Figure 6B), suggesting that these mutants accumulate DNA damage probably during normal DNA replication. It is important to note that these mutants are defective in Swi1-Swi3 complex formation (Figure 2A). Interestingly, *swi3-E39* (Class III) cells failed to show a significant increase in spontaneous DNA damage foci (Figure 6B), suggesting that these cells are proficient in normal DNA replication. Since *swi3-E39* (Class III) is largely defective in the recovery of broken replication forks (Figure 5A), the results suggest that Swi3 has a specific function in facilitating repair of broken forks. We have also monitored Rad22-YFP in single-point *swi3* mutants and found that *swi3-D84H* (E31), *swi3-W95R* (E40), *swi3-Y111C* (E68) and *swi3-L112R* (NBT7) cells have greatly increased DNA repair foci formation (Figure 6B). All these mutants were defective in Swi1-Swi3 complex formation, suggesting the importance of the Swi1–Swi3 complex in suppression of spontaneous DNA damage during unperturbed DNA replication.

### Sister chromatid cohesion abnormalities in *swi3* mutants

**Figure 7:**
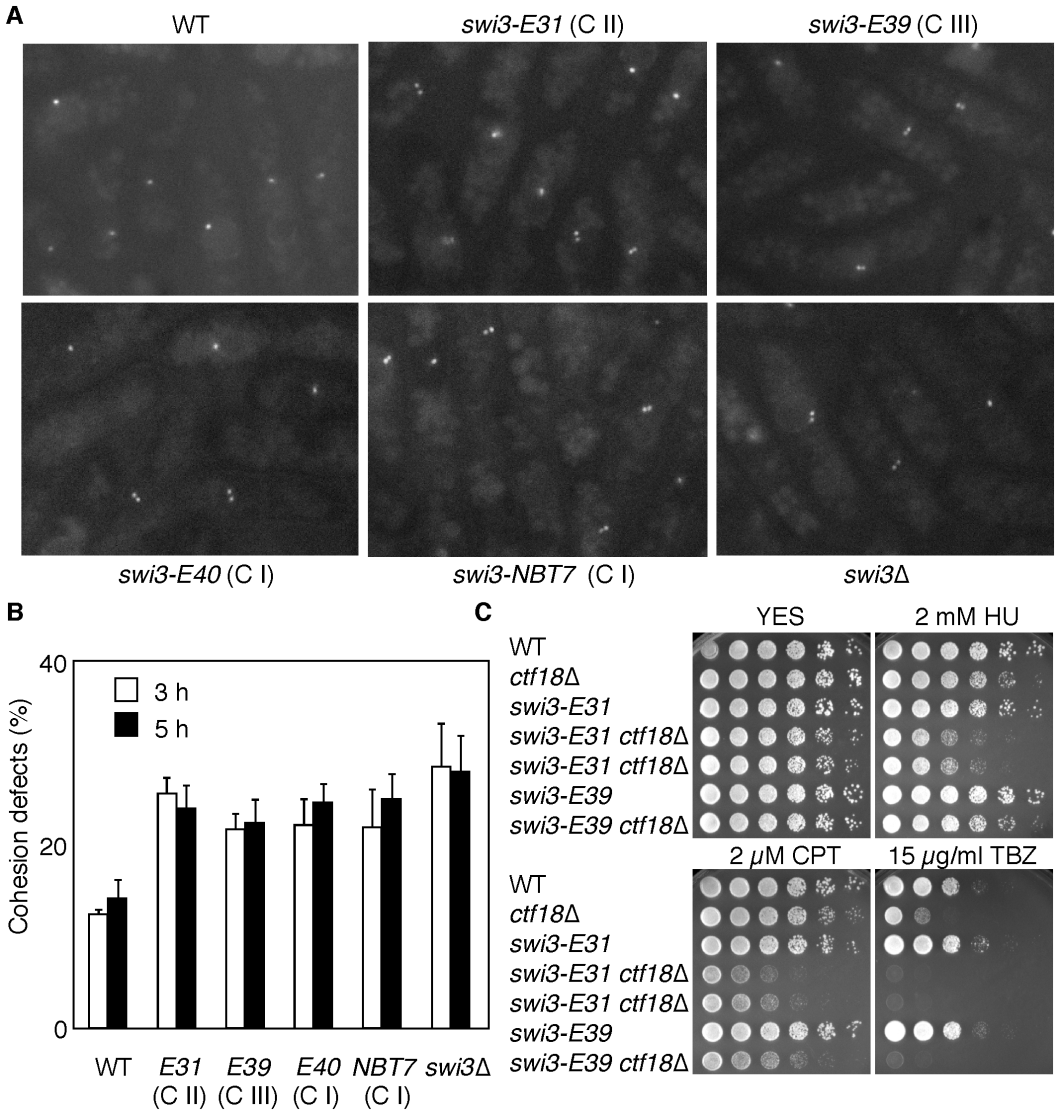
Effects of *swi3* mutations on sister chromatid cohesion. (**A**) Cells of the indicated genotypes were grown to mid-log phase and incubated at 20°C for 3 and 5 h to obtain prophase/metaphase cells. All cells contain the *nda3-KM311* mutation and LacO repeats near centromere 1 and express LacI-GFP-NLS. Representative images at 5 h are shown for cells of indicated genotypes. (**B**) Quantification of prophase/metaphase cells that had two GFP foci shown in A. At least 200 cells were counted for each strain. Error bars correspond to the standard deviations obtained from at least three independent experiments. (**C**) Five-fold serial dilutions of cells of the indicated genotypes were incubated on YES agar medium supplemented with the indicated amounts of HU, CPT, and TBZ for 3 to 5 days at 32°C. *swi3-E31* and *swi3-E39* has synergistic genetic interaction with *ctf18*Δ in CPT and TBZ sensitivities. However, *swi3-E31* but not *swi3-E39* had additive genetic effect with *ctf18*Δ in HU sensitivity, strengthening the idea that *swi3-E39* is proficient in the Cds1-dependent replication checkpoint. Representative images of repeat experiments are shown.

We have previously found that Swi1 and Swi3 are required for proper establishment of sister chromatid cohesion [37]. Therefore, we examined the effect of *swi3* mutations on sister chromatid cohesion. To monitor cohesion defects in *swi3* mutants, we used a strain that has the bacterial *LacO* tandem repeat sequences inserted at the *lys1* locus located in the vicinity of the centromere on chromosome I. This strain is engineered to express the LacI repressor fused to GFP-NLS, which is recruited to *LacO* repeat sequences, allowing us to visualize centromere 1 [37], [40]. If sister chromatids are properly adhered to one another, the GFP signal should resolve as a single focus in the nuclei until cells enter anaphase when cells separate two sister chromatids. However, if sister chromatids are prematurely separated, two distinct GFP foci would occur before cells enter anaphase. Using this system, we determined the effect of *swi3* mutations on cohesion at the centromere region. For synchronization, we used *nda3-KM311* cold-sensitive background to arrest cells at prophase/metaphase by culturing cells at 20°C [41]. Because sister chromatids are still attached to one another at prophase/metaphase, the majority of wild-type cells showed a single centromere focus in nuclei (Figure 7A). In contrast, the experiments revealed a significant increase in the number of nuclei with two foci in *swi3-NBT7*, *swi3-E31*, *swi3-E39* and *swi3-E40* cells (Figure 7A and 7B). This indicates that these mutants have a defect in efficient establishment of sister chromatid cohesion. Moreover, considering the fact that *swi3-E39* has defects in replication recovery after fork breakage but not in Cds1 activation (Figure 5A and 6A), our results are consistent with the notion that the checkpoint role of Swi3 is not sufficient for proper establishment of sister chromatid cohesion. The data also suggest that *swi3-E39* has a defect in a specific function that is required to coordinate with cohesion processes.

We have previously shown that *swi3*Δ is synthetically lethal with deletion of *ctf18*, which encode the largest subunit of an alternative replication factor C complex (RFC^Ctf18^) required for establishment of sister chromatid cohesion [37], [42], [43]. *swi3-NBT7* and *swi3-E40* were found to be synthetically lethal with *ctf18*Δ (data not shown). This is consistent with the fact that these mutants displayed phenotypes similar to those of *swi3*Δ cells. Although *swi3-E31 ctf18*Δ and *swi3-E39 ctf18*Δ cells were viable, these double mutants were much more sensitive to CPT compared to either single mutant (Figure 7C). Importantly, although *swi3-E31* and *swi3-E39* were not sensitive to 15 µg/ml of thiabendazole (TBZ), *swi3-E31 ctf18*Δ and *swi3-E39 ctf18*Δ showed TBZ hypersensitivity (Figure 7C). TBZ sensitivity is found among mutants that affect general sister chromatid cohesion and segregation [44], [45], [46], [47]. Therefore, these results strengthen the fact that the cohesion function of Swi3 is defective in *swi3-E31* and *E39*.

## Discussion

Programmed fork pausing and replication termination events near the mating-type (*mat1*) locus are needed to create an imprint and initiate a gene conversion event that switches mating-type in fission yeast [48], [49]. These events require *swi1* ^+^ and *swi3* ^+^ genes [48]. Since mutations in these genes were found to be synthetically lethal with a mutation in DNA polymerase α, the role of *swi1* ^+^ and *swi3* ^+^ in DNA replication was proposed [48]. Accordingly, Swi1 and Swi3 have been identified and shown to form a stable complex that plays critical roles in stabilization of replication forks, activation of the replication checkpoint, and coordination of leading- and lagging-strand DNA synthesis [19], [20], [23], [24], [48], [50], [51]. In addition, Swi1 and Swi3 are required for proper establishment of sister chromatid cohesion [37]. However, the molecular mechanisms by which Swi1 and Swi3 stabilize replication forks and contribute to various replication-associated events remain elusive. Therefore, in this report, as an initial step toward dissecting the molecular pathways that require the Swi1–Swi3 replication fork protection complex, we performed mutational analyses of Swi3. Accordingly, we found separation-of-function mutations that led us to the conclusion that Swi3 utilizes different pathways to regulate the replication checkpoint and replication-dependent sister chromatid cohesion.

### Roles of Swi1–Swi3 complex as a checkpoint mediator

Our investigation suggest that the central conserved region of Swi3 is essential for interacting with Swi1 (Figure 2 and 3 and Table 1) and that Swi1–Swi3 complex formation is required for S-phase stress response (Figures 1A and 1B). Mutations that abolish Swi1–Swi3 complex formation sensitize cells to many different S-phase stressing agents. *swi3-NBT7*, *E40* and *E31* mutants (Classes I and II), all of which have a defect in Swi1-Swi3 complex formation, showed significant sensitivity to HU, MMS and CPT (Figure 1A). HU and MMS cause an arrest of the replication fork, which in turn activates the Cds1-dependent replication checkpoint. Consistently, *swi3-E31*, *swi3-NBT7* and *E40* had impaired Cds1 activity (Figure 6A) and had significant defects in resumption of replication after HU treatment (Figure 5B). Since replication resumption from HU arrest requires Cds1 [9], [13], [19], our data suggest that Swi1–Swi3 complex formation plays a critical role in activation of the replication checkpoint and stabilization of stalled replication forks in response to HU. In addition to the checkpoint defect in *swi3-NBT7*, *E40* and *E31*, these cells showed strong accumulation of spontaneous Rad22 DNA repair foci, indicative of DNA damage (Figure 6B). Consistently, when we examined single-point mutants defective in Swi1–Swi3 complex formation, cells showed dramatic accumulation of Rad22-YFP DNA repair foci in the absence of genotoxic agents (Figure 6B and Table 1). Therefore, although there is a possibility that these mutants might not be solely defective in Swi1–Swi3 complex formation, our results are consistent with the notion that Swi1–Swi3 complex formation is also important to prevent DNA damage, probably during normal DNA replication.

Intriguingly, all mutations affecting Swi1–Swi3 complex formation were located in one of the putative alpha helices found in the central conserved domain (Figure 3B, Domain II), suggesting that such alpha helix structures play an important role in protein-protein interaction. Interestingly, Swi3-E68 (K47N, Y111C) retained the ability to interact with Swi1 (Figure 2A), and corresponding mutant cells were sensitive to CPT, but not HU and MMS (Figure 1A). In contrast, *swi3-Y111C* mutant cells were highly sensitive to HU, MMS and CPT (Figure 1B), and the Swi3-Y111C protein failed to interact with Swi1 (Figure 2B). This indicates that the K47N mutation alleviates the defect of *swi3-E68* cells in Swi1–Swi3 complex formation and restores tolerance to HU and MMS, agents that activate the replication checkpoint. These results further support the idea that Swi1–Swi3 complex formation is essential for its function as a mediator of the replication checkpoint.

### Roles of Swi3 in the recovery of broken replication forks

**Figure 8:**
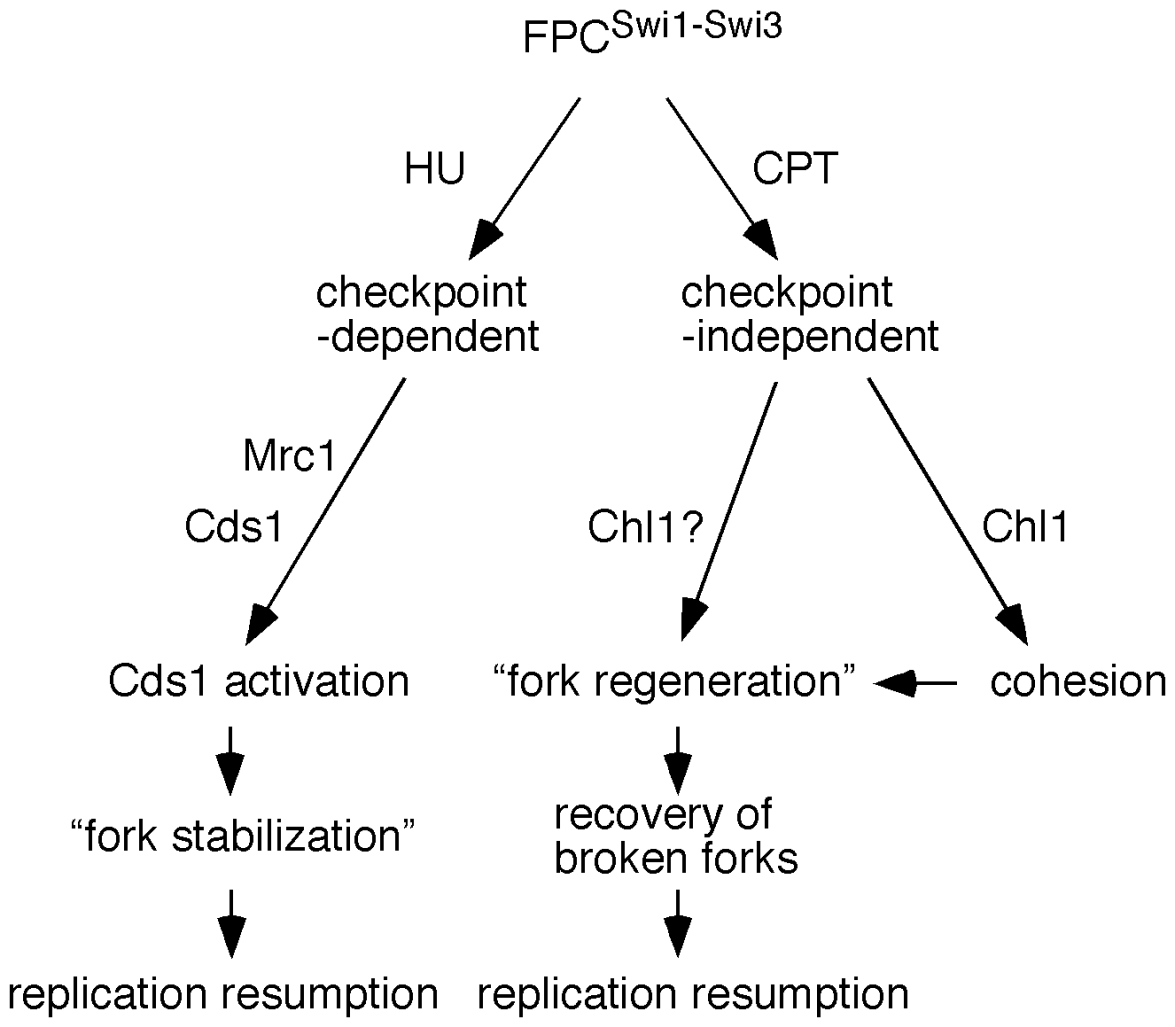
Models for Swi1–Swi3 dependent preservation of genomic integrity in *S. pombe*. Swi1–Swi3 complex is involved in both checkpoint-dependent and -independent pathways to maintain genomic integrity. Swi1–Swi3 regulates Mrc1 and Cds1 to promote checkpoint activation and fork stabilization in response to HU-dependent fork arrest. Swi1–Swi3 uses a checkpoint-independent mechanism to regenerate broken replication forks when cells are treated with CPT. Swi1–Swi3 may regulate Chl1 to promote efficient establishment of sister chromatid cohesion, which might also be involved in fork regeneration.

It is important to note that some of the *swi3* mutants (Class III mutants: *swi3-E1, E39*, *E59* and *E68*) were only sensitive to CPT, which causes replication fork breakage (Figure 1). In these mutants, Swi1–Swi3 complex formation was unaffected, and cells failed to show significant HU sensitivity (Figures 1A, 2A and Table 1). Consistently, all Class III mutants had robust Cds1 activation in response to HU (Figure 6A and Table 1). In addition, *swi3-E39* cells were able to normally resume DNA replication after HU-dependent fork arrest (Figure 5B). Since *swi3-E39* cells were not able to recover damaged replication forks provoked by CPT (Figure 5A), our results suggest that Swi3 regulates at least two separate pathways. The first pathway is checkpoint-dependent, which is to promote Cds1 activation and stabilize stalled replication fork in response to HU-dependent fork arrest (Figure 8). The second pathway is to promote efficient DNA replication and/or replication recovery after CPT-dependent fork breakage, which is independent of the Cds1-dependent replication checkpoint (Figure 8). This model is consistent with the previous study that reported the role of Swi3 in survival of MMS, which is also independent of Cds1- and Chk1-mediated checkpoints [23]. It has been known that Cds1 is involved in replication fork stabilization in response to HU in *S. pombe* [9], [13], [19]. It has also been reported in *S. cerevisiae* that Rad53 (Cds1 homolog) is required to prevent accumulation of unusual DNA structures at the replication fork in response to fork arrest induced by HU or MMS [14], [16], [17]. Since Swi1–Swi3 is required for the chromatin association of Mrc1, which is essential for Cds1 activation [52], Swi1–Swi3 may regulate the replication checkpoint pathway by recruiting Mrc1 to activate Cds1 and promote fork stabilization in response to HU (Figure 8). However, in the presence of CPT, Cds1 is dispensable when cells restore broken replication forks (Figure 5A). Therefore, fork stabilization function of Cds1/Rad53 may be important when the fork is arrested by dNTP depletion (HU) or alkylation of template DNA (MMS), and this function is checkpoint-dependent. However, when cells are treated with CPT, replication forks must be recovered by a different mechanism that utilizes Swi1–Swi3, but is independent of the replication checkpoint (Figure 8). It is possible that Swi1–Swi3 facilitates efficient repair of broken replication forks, although further investigation is needed to address this possibility. It is also feasible that Swi1–Swi3 promotes DNA replication after DSBs at forks have been repaired. Therefore, we propose a model in which Swi1–Swi3 is involved in at least two processes during fork recovery. First, Swi1–Swi3 is required to resume arrested replication fork in a replication checkpoint-dependent manner. This process can be referred to as “fork stabilization” (Figure 8). Second, Swi1–Swi3 may also be important to re-capture replication fork and/or re-assemble replisome components when forks are broken. This “fork regeneration process” is independent of the replication checkpoint (Figure 8). Our results are consistent with the idea that Class I and II mutants have defects in both “fork stabilization” and “fork regeneration” processes, while *swi3-E39* mutant (Class III) is proficient in “fork stabilization” but defective in “fork regeneration”. In budding yeast, it is proposed that Tof1-Csm3-Mrc1 form a “fork pausing complex”, which is required to stabilize stalled replication forks [28]. In this model, the fork pausing complex is involved in coupling of polymerases and helicases at stalled replication forks. However, in *S. pombe*, Swi1–Swi3 (Tof1-Csm3 homolog) only weakly associates with Mrc1, while the interaction between Swi1 and Swi3 is tight [20], [52]. In addition, our data strongly support the idea that Swi1–Swi3 also plays a role in fork-recapture and/or -reassembly when forks are actually broken. Therefore, we prefer the model in which Swi1–Swi3 functions as a “fork protection complex” that promotes both fork-stabilization and fork-regeneration processes in response to various genotoxic agents (Figure 8).

### Roles of Swi3 in replication-coupled sister chromatid cohesion

The present studies revealed that a separation-of-function mutation of Swi3, which render cells sensitive specifically to CPT, also caused sister chromatid cohesion defects comparable to *swi3* deletion mutants. This mutant (*swi3-E39*) also had defects in recovery of broken replication forks but not in resumption of arrested forks (Figure 5), the latter of which is dependent on the Cds1-dependent replication checkpoint. It has been thought that proteins involved in replication checkpoint safeguard sister chromatid cohesion [53]. While this is true, our present results are consistent with the notion that the checkpoint and cohesion roles of Swi3 are separable, and that the replication checkpoint function of Swi3 is not sufficient for cohesion process. Intriguingly, fork-regeneration function of Swi3 is coupled with sister chromatid cohesion (Figure 8). Therefore, we propose that the replication checkpoint and chromosome cohesion function in separate pathways. We also propose that Swi1–Swi3 has a key role in replication-coupled sister chromatid cohesion established at the replication fork. Consistently, we have shown that Timeless interacts with cohesin subunits in human cells [35]. Moreover, Timeless downregulation led to dissociation of cohesin subunits from chromatin and defects in sister chromatid cohesion in human cells [35]. Interestingly, we have also demonstrated in both *S. pombe* and human cells that Swi1–Swi3^Timeless-Tipin^ acts together with Chl1^ChlR1^, a DNA helicase known to be required for establishment of sister chromatid cohesion [35], [37]. Therefore, we suggest that Swi1–Swi3^Timeless-Tipin^ and Chl1^ChlR1^are in the same pathway to control fork regeneration and cohesion processes (Figure 8).

Recent studies have shown the role of sister chromatid cohesion in the repair of DSBs [54], [55]. Therefore, we also speculate that improper cohesion in the absence of Swi3 can affect efficient repair of DSBs at replication forks when cells are treated with camptothecin. Therefore, it is possible that Swi1–Swi3 facilitates sister chromatid cohesion to promote efficient recapture of the fork during recombination processes, which also contribute to the regeneration of replication forks (Figure 8).

## Materials and Methods

### General Techniques

The methods used for genetic and biochemical analyses of fission yeast have been described previously [56], [57]. PCR amplification of DNA was done using EX taq DNA polymerase (TaKaRa, Ohtsu, Japan). Accurate PCR reactions were confirmed by DNA sequencing analyses. Western blotting, Cds1 kinase assay, and drug sensitivity assays were performed as described in our earlier studies [37], [58]. For immunoblotting, Myc, TAP, and FLAG fusion proteins were probed with the anti-c-Myc 9E10 monoclonal antibody (Covance, Berkeley, CA), PAP (Peroxidase Anti-Peroxidase Soluble Complex antibody) (Sigma-Aldrich, St. Louis, MO), and the anti-FLAG M2 monoclonal antibody (Sigma-Aldrich), respectively. TAT-1 [59] was used to detect tubulin. Microscopic analyses of green fluorescent protein (GFP) and yellow fluorescent proteins (YFP) were performed using Olympus PROVIS AX70 microscope equipped with a Retiga EXi camera (QImaging, Surrey, BC, Canada). Images were acquired with Ivision software (BioVision Technologies, Exton, PA).

### Plasmids

Genomic DNA was isolated from *S. pombe* cells containing the *swi3-TAP-kanMX6* gene [20]. The 1.7 kb *swi3-TAP* genomic fragment including the *swi3* promoter region was amplified by PCR from this genomic DNA preparation, and subsequently cloned into the XbaI/KpnI site of pJK148 [60] to generate pJK148-swi3-TAP. The 1.3 kb mutant *swi3-5FLAG* fragments were amplified by PCR from genomic DNA prepared from *swi3* mutants, and cloned into the XbaI/BamHI site of pJK148 to generate pJK148-swi3-5FLAG. The 1.5 kb NotI-BglII fragment containing a C-terminal *rad22* region fused with *YFP* cDNA [19], [61] was introduced into the NotI/BamHI site of pJK210 [60], resulting in pJK210-rad22-YFP-CT.

### *S. pombe* strains

The *S. pombe* strains used in this study were constructed using standard techniques [56], and their genotypes are listed in Supplementary Table S1. *swi1-13Myc* (*swi1-13Myc-hphMX6*), *swi3-13Myc* (*swi3-13Myc-hphMX6*) and *ctf18*Δ (*ctf18*::*hphMX6*) were generated by a one-step marker switch method [62] using the *swi1-13Myc-kanMX6*, *swi3-13Myc-kanMX6* and *ctf18*::*kanMX6* strains, respectively. Single-point *swi3* mutants were generated by Kunkel site-directed mutagenesis [63] in pJK148-swi3-TAP, and integrated at the *leu1* locus of the *swi3*::*kanMX6 swi1-3FLAG-kanMX6* strain. To visualize Rad22-YFP in *swi3* mutants, pJK210-Rad22YFP-CT was integrated at the *rad22* locus of the *swi3* mutant strains. To monitor cohesion defects, pJK148-swi3 (wild-type or mutants)-5FLAG was integrated at *leu1* locus of an *S. pombe* strain containing *nda3-KM311*, *swi3*::*KanMX6*, *lys1* ^+^:*LacO* repeat and *his7* ^+^:*GFP-LacI-NLS*.

Mutations and epitope-tagged genes have previously been described for *swi1*Δ (*swi1*::*kanMX6*) [19]; *swi1-13Myc* (*swi1-13Myc-kanMX6*), *swi1-3FLAG* (*swi1-3FLAG-kanMX6*), *swi3*Δ (*swi3*::*kanMX6*), *swi3-TAP* (*swi3-TAP*-*kanMX6*), *swi3-3FLAG* (*swi3-3FLAG-kanMX6*), *swi3-13Myc* (*swi3-13Myc-kanMX6*) [20], *cds1*Δ (*cds1*::*kanMX6*), *chk1*Δ (*chk1*::*kanMX6*), *rad3*Δ (*rad3*::*kanMX4*), *ctf18*Δ (*ctf18*::*kanMX6*) [37], *rad26*Δ (*rad26*::*ura4* ^+^) [64], *nda3-KM311* [41], and *lys1* ^+^-*LacO* repeat *his7* ^+^-*dis1promoter*-*GFP-LacI-NLS* [40].

### Isolation of *swi3* mutants

Genomic DNA was isolated from *S. pombe* cells containing the *swi3-5FLAG-kanMX6* gene [20]. The 2.9 kb *swi3-5FLAG-kanMX* genomic fragment was amplified from this genomic DNA preparation by PCR, and subsequently cloned into the AdhI site of pBluescript II TKS (+) [65] to generate the pTKS-swi3-5FLAG-kanMX construct. Error-prone PCR was performed using five- and threefold higher than recommended concentrations of EX taq DNA polymerase and dNTPs, respectively. The wild-type *swi3* ^+^ gene was replaced with the mutagenized *swi3-5FLAG-kanMX6* gene at the *swi3* locus by a standard transformation method. Kanamycin-resistant colonies were isolated and their growth was examined to select for hydroxyurea- and camptothecin-sensitive mutants. This method generated eight *swi3* mutants, which are designated *swi3-E1*, *swi3-E10*, *swi3-E31*, *swi3-E39*, *swi3-E40*, *swi3-E42*, *swi3-E59* and *swi3-E68*. We also isolated the *swi3-NBT7* mutant by selecting for mating-type switching defective mutants.

### Precipitation of TAP and FLAG-tagged proteins

Precipitation of TAP-tagged proteins were performed using immunoglobulin G-Sepharose beads (GE Healthcare, Piscataway, NJ) as previously described [37]. For precipitation of FLAG-tagged proteins, cells expressing FLAG-fusion proteins were cultured in YES medium and collected when an optical density of 1.2 at 600 nm was reached. Cells were then lysed with glass beads in lysis buffer A {50 mM Tris-HCl (ph 8.0), 150 mM NaCl, 0.1% NP-40, 10% glycerol, 50 mM NaF, 1 mM Na_3_VO_4_, 5 mM EDTA, 5 mM *N*-methylmaleimide, 1 µM microcyctin, 0.1 µM okadaic acid, 0.2 mM *p*-4-amidoinophenyl-methane sulfonyl fluoride hydrochloride monohydrate (*p*-APMSF) and Roche complete EDTA-free protease inhibitor cocktail} using a FastPrep cell disrupter (Qbiogene, Irvine, CA) for two cycles of 20 seconds each at speed 6, with a one-minute interval on ice between the two cycles. Protein extracts were clarified by centrifugation at 13,000 rpm in an Eppendorf microcentrifuge 5415D for 10 min at 4°C, mixed with anti-FLAG M2 agarose (Sigma-Aldrich) and incubated for 2 hr at 4°C. The agarose beads were collected and washed three times in lysis buffer A. Proteins associated with the beads were analyzed by Western blotting.

### Pulsed-field gel electrophoresis (PFGE)

Exponentially growing cells were treated with the indicated amount of camptothecin (CPT) or hydroxyurea (HU) for 3 h at 30°C, and then they were washed and released into fresh YES medium. Cells were collected at the indicated times, and chromosomal DNA samples were prepared in agarose plug and analyzed with CHEF-DRII system (Bio-Rad) as previously described [37], [58].

### Detection of Rad22-YFP DNA repair foci

Cells expressing Rad22-YFP foci from its own promoter were grown at 25°C in YES liquid medium until mid-log phase, and then Rad22-YFP localization was analyzed as previously described [37], [58]. At least 200 cells were counted for each strain in each experiment.

### Chromosome cohesion assay

Chromosome cohesion assay was performed as described previously [37]. We used a cold-sensitive *nda3-K311* strain harboring bacterial *LacO* tandem repeat sequences inserted in the vicinity of the centromere on chromosome 1 [40]. This strain is engineered to express the LacI repressor fused to GFP-nuclear localization signal (NLS), which is recruited to *LacO* repeat sequences, allowing to visualize the centromere 1 [40]. The *nda3-K311* cells were grown to mid-log phase at 30°C and shifted to a restrictive temperature, 20°C. At the indicated time, GFP foci were monitored and imaged. Quantification of GFP foci has been performed at least three times, and at least 200 cells were counted for each strain in each experiment.

## Supporting Information

Table S1 *S. pombe* strains used in this study.
(0.05 MB DOC)
