# Supplementary material for: Checkpoint-Dependent and -Independent Roles of Swi3 in Replication Fork Recovery and Sister Chromatid Cohesion in Fission Yeast
Source: PLoS One. 2010 Oct 12;5(10):e13379. doi: 10.1371/journal.pone.0013379 (PMC2953522; doi:10.1371/journal.pone.0013379)
Supplement: Table S1 — S. pombe strains used in this study. (0.05 MB DOC) [file pone.0013379.s001.doc]

**Supplemental Table S1.** *S. pombe* strains used in this study

Strain Genotype Source

Y0001 *h*^-^ Laboratory Stock

Y0002 *h*^+^ Laboratory Stock

Y0172 *h*^-^ *swi1-13Myc*:*KanMX6* Noguchi et al, 2004

Y0211 *h*^-^ *swi1*::*kanMX6* Noguchi et al, 2003

Y0282 *h*^-^ *rad26*::*ura4*^+^ Laboratory Stock

Y0286 *h*^-^ *swi1*::*kanMX6 rad26*::*ura4*^+^ Noguchi et al, 2003

Y0613 *h*^-^ *swi1-3FLAG*:*KanMX6* Noguchi et al, 2004

Y0633 *h*^+^ *swi3*::*KanMX6* Noguchi et al, 2004

Y0636 *h*^-^ *swi3-3FLAG*:*KanMX6* Noguchi et al, 2004

Y0642 *h*^-^ *swi3-13Myc*:*KanMX6* Noguchi et al, 2004

Y0674 *h*^-^ *swi3*::*KanMX6* *rad22-YFP*:*KanMX6* Noguchi et al, 2004

Y0800 *h*^-^ *swi3*::*kanMX6 rad26*::*ura4*^+^ This study

Y1034 *h*^+^ *swi3-E1-3FL*:*KanMX6* This study

Y1038 *h*^+^ *swi3-E10-3FL*:*KanMX6* This study

Y1044 *h*^+^ *swi3-E31-3FL*:*KanMX6* This study

Y1045 *h*^+^ *swi3-E39-3FL*:*KanMX6* This study

Y1046 *h*^+^ *swi3-E40-3FL*:*KanMX6* This study

Y1047 *h*^+^ *swi3-E42-3FL*:*KanMX6* This study

Y1049 *h*^+^ *swi3-E59-3FL*:*KanMX6* This study

Y1052 *h*^+^ *swi3-E68-3FL*:*KanMX6* This study

Y1551 *h*^+^ *chk1*::*KanMX6* Laboratory Stock

Y1565 *h*^+^ *cds1*::*KanMX6* Laboratory Stock

Y1569 *h*^+^ *rad3*::*KanMX4* Laboratory Stock

Y1633 *h*^+^ *swi1-13myc*:*hphMX6* This study

Y1656 *h*^+^ *swi1-13myc*:*hphMX6 swi3-E1-3FLAG*:*KanMX6*  This study

Y1658 *h*^+^ *swi1-13myc*:*hphMX6 swi3-E10-3FLAG*:*KanMX6* This study

Y1660 *h*^-^ *swi1-13myc*:*hphMX6 swi3-E31-3FLAG*:*KanMX6* This study

Y1662 *h*^-^ *swi1-13myc*:*hphMX6* *swi3-E39-3FLAG*:*KanMX6* This study

Y1664 *h*^-^ *swi1-13myc*:*hphMX6 swi3-E40-3FLAG*:*KanMX6* This study

Y1666 *h*^-^ *swi1-13myc*:*hphMX6 swi3-E42-3FLAG*:*KanMX6* This study

Y1668 *h*^-^ *swi1-13myc*:*hphMX6 swi3-E59-3FLAG*:*KanMX6* This study

Y1670 *h*^-^ *swi1-13myc*:*hphMX6 swi3-E68-3FLAG*:*KanMX6* This study

Y1672 *h*^-^ *swi1-13myc*:*hphMX6* *swi3-3FLAG*:*KanMX6* This study

Y1800 *nda3-KM311* *swi3*::kanMX6 *lys1*^+^:*lacOrepeat his7*^+^:*dis1promoter-GFP-LacI-NLS* Ansbach et al, 2008

Y1808 *h*^+^ *swi3-13myc*:*hphMX6* This study

Y1986 *h*^+^ *swi1-13myc*:*hphMX6* *swi3-NBT7-3FLAG*:*KanMX6* This study

Y2247 *h*^-^ *swi1-3FLAG*:*KanMX6* *swi3*::*KanMX6* *leu1-32*::*leu1*^+^:*swi3-TAP* This study

Y2249 *h*^-^ *swi1-3FLAG*:*KanMX6* *swi3*::*KanMX6* *leu1-32*::*leu1*^+^:*swi3-D84H-TAP* This study

Y2251 *h*^-^ *swi1-3FLAG*:*KanMX6* *swi3*::*KanMX6* *leu1-32*::*leu1*^+^:*swi3*-*F171L*-*TAP* This study

Y2253 *h*^-^ *swi1-3FLAG*:*KanMX6* *swi3*::*KanMX6* *leu1-32*::*leu1*^+^:*swi3*-*N17I*-*TAP* This study

Y2255 *h*^-^ *swi1-3FLAG*:*KanMX6* *swi3*::*KanMX6* *leu1-32*::*leu1*^+^:*swi3*-*W95R*-*TAP* This study

Y2256 *h*^-^ *swi1-3FLAG*:*KanMX6* *swi3*::*KanMX6* *leu1-32*::*leu1*^+^:*swi3*-*Y111C*-*TAP* This study

Y2257 *h*^-^ *swi1-3FLAG*:*KanMX6* *swi3*::*KanMX6* *leu1-32*::*leu1*^+^:*swi3*-*K78R*-*TAP* This study

Y2258 *h*^-^ *swi1-3FLAG*:*KanMX6* *swi3*::*KanMX6* *leu1-32*::*leu1*^+^:*swi3*-*K47N*-*TAP* This study

Y2260 *h*^-^ *swi1-3FLAG*:*KanMX6* *swi3*::*KanMX6* *leu1-32*::*leu1*^+^:*swi3*-*L112R*-*TAP* This study

Y2261 *h*^-^ *swi1-3FLAG*:*KanMX6* *swi3*::*KanMX6* *leu1-32*::*leu1*^+^:*swi3*-*Y111N*-*TAP* This study

Y2263 *h*^-^ *swi1-3FLAG*:*KanMX6* *swi3*::*KanMX6* *leu1-32*::*leu1*^+^:*swi3*-*R124L*-*TAP* This study

Y2384 *h*^-^ *swi1-3FLAG*:*KanMX6* *swi3*::*KanMX6* *leu1-32*::*leu1*^+^:*swi3-TAP* *rad22*-*YFP*:*ura4*^+^ This study

Y2385 *h*^-^ *swi1-3FLAG*:*KanMX6* *swi3*::*KanMX6* *leu1-32*::*leu1*^+^:*swi3-D84H-TAP rad22*-*YFP*:*ura4*^+^ This study

Y2386 *h*^-^ *swi1-3FLAG*:*KanMX6* *swi3*::*KanMX6* *leu1-32*::*leu1*^+^:*swi3*-*F171L*-*TAP rad22*-*YFP*:*ura4*^+^ This study

Y2387 *h*^-^ *swi1-3FLAG*:*KanMX6* *swi3*::*KanMX6* *leu1-32*::*leu1*^+^:*swi3*-*N17I*-*TAP* *rad22*-*YFP*:*ura4*^+^ This study

Y2388 *h*^-^ *swi1-3FLAG*:*KanMX6* *swi3*::*KanMX6* *leu1-32*::*leu1*^+^:*swi3*-*W95R*-*TAP* *rad22*-*YFP*:*ura4*^+^ This study

Y2389 *h*^-^ *swi1-3FLAG*:*KanMX6* *swi3*::*KanMX6* *leu1-32*::*leu1*^+^:*swi3*-*Y111C*-*TAP* *rad22*-*YFP*:*ura4*^+^ This study

Y2390 *h*^-^ *swi1-3FLAG*:*KanMX6* *swi3*::*KanMX6* *leu1-32*::*leu1*^+^:*swi3*-*K78R*-*TAP* *rad22*-*YFP*:*ura4*^+^ This study

Y2391 *h*^-^ *swi1-3FLAG*:*KanMX6* *swi3*::*KanMX6* *leu1-32*::*leu1*^+^:*swi3*-*K47N*-*TAP* *rad22*-*YFP*:*ura4*^+^ This study

Y2392 *h*^-^ *swi1-3FLAG*:*KanMX6* *swi3*::*KanMX6* *leu1-32*::*leu1*^+^:*swi3*-*L112R*-*TAP* *rad22*-*YFP*:*ura4*^+^ This study

Y2393 *h*^-^ *swi1-3FLAG*:*KanMX6* *swi3*::*KanMX6* *leu1-32*::*leu1*^+^:*swi3*-*Y111N*-*TAP* *rad22*-*YFP*:*ura4*^+^ This study

Y2394 *h*^-^ *swi1-3FLAG*:*KanMX6* *swi3*::*KanMX6* *leu1-32*::*leu1*^+^:*swi3*-*R124L*-*TAP*  *rad22*-*YFP*:*ura4*^+^ This study

Y3031 *h*^-^ *swi3-E31-3FL*:*KanMX6* *swi1-13myc*:*hphMX6* *rad22-YFP*:*ura4*^+^ This study

Y3033 *h*^-^ *swi3-E39-3FL*:*KanMX6* *swi1-13myc*:*hphMX6* *rad22-YFP*:*ura4*^+^ This study

Y3035 *h*^-^ *swi3-E40-3FL*:*KanMX6* *swi1-13myc*:*hphMX6* *rad22-YFP*:*ura4*^+^ This study

Y3037 *h*^-^ *swi3-3FL*:*KanMX6* *swi1-13myc*:*hphMX6* *rad22-YFP*:*ura4*^+^ This study

Y3039 *h*^+^ *swi3-NBT7-3FL*:*KanMX6* *swi1-13myc*:*hphMX6* *rad22-YFP*:*ura4*^+^ This study

Y3074 *h*^+^ *ctf18*::*hphMX6* This study

Y3129 *h*^+^ *swi3-NBT7-3FL*:*KanMX6* This study

Y3131 *h*^+^ *ctf18*::*hphMX6* *swi3-E31-3FL*:*KanMX6* This study

Y3133 *h*^+^ *ctf18*::*hphMX6 swi3-E39-3FL*:*KanMX6* This study

Y3175 *nda3-KM311* *leu1-32*::*leu1*^+^:*swi3-E31-5FLAG* *swi3*::kanMX6 *cen1-GFP* This study

Y3177 *nda3-KM311* *leu1-32*::*leu1*^+^:*swi3-E39-5FLAG* *swi3*::kanMX6 *cen1-GFP* This study

Y3179 *nda3-KM311* *leu1-32*::*leu1*^+^:*swi3-E40-5FLAG* *swi3*::kanMX6 *cen1-GFP* This study

Y3181 *nda3-KM311* *leu1-32*::*leu1*^+^:*swi3-NBT7-5FLAG* *swi3*::kanMX6 *cen1-GFP* This study

Y3183 *nda3-KM311* *leu1-32*::*leu1*^+^:*swi3-5FLAG* *swi3*::kanMX6 *cen1-GFP* This study

* All strains are also *leu1-32* and *ura4-D18*

** Y1800, Y3175, Y3177, Y3179, Y3181, and Y3183 have *his3-D1*

*****The actual genotype of *cen1-GFP* is *lys1*^+^:*lacOrepeat his7*^+^:*dis1promoter-GFP-LacI-NLS*
